# Changes in the Expression of Pre-Replicative Complex Genes in hTERT and ALT Pediatric Brain Tumors

**DOI:** 10.3390/cancers12041028

**Published:** 2020-04-22

**Authors:** Aurora Irene Idilli, Francesca Pagani, Emanuela Kerschbamer, Francesco Berardinelli, Manuel Bernabé, María Luisa Cayuela, Silvano Piazza, Pietro Luigi Poliani, Emilio Cusanelli, Maria Caterina Mione

**Affiliations:** 1 Department of Cellular, Computational and Integrative Biology–CIBIO, University of Trento, Via Sommarive 9, 38123 Trento, Italy; 2 Pathology, Department of Molecular and Translational Medicine, University of Brescia, 25123 Brescia, Italy; 3 Department of Science, University of Rome “Roma Tre”, 00145 Rome, Italy; 4 Telomerase, Cancer and Aging, Department of Surgery, Instituto Murciano de Investigación Biosanitaria-Arrixaca, 30005 Murcia, Spain

**Keywords:** pediatric brain tumors, ALT, telomere maintenance mechanisms, pre-replicative complex, heterochromatin, zebrafish, C-circles

## Abstract

*Background*: The up-regulation of a telomere maintenance mechanism (TMM) is a common feature of cancer cells and a hallmark of cancer. Routine methods for detecting TMMs in tumor samples are still missing, whereas telomerase targeting treatments are becoming available. In paediatric cancers, alternative lengthening of telomeres (ALT) is found in a subset of sarcomas and malignant brain tumors. ALT is a non-canonical mechanism of telomere maintenance developed by cancer cells with no-functional telomerase. *Methods*: To identify drivers and/or markers of ALT, we performed a differential gene expression analysis between two zebrafish models of juvenile brain tumors, that differ only for the telomere maintenance mechanism adopted by tumor cells: one is ALT while the other is telomerase-dependent. *Results*: Comparative analysis of gene expression identified five genes of the pre-replicative complex, *ORC4, ORC6, MCM2*, *CDC45* and *RPA3* as upregulated in ALT. We searched for a correlation between telomerase levels and expression of the pre-replicative complex genes in a cohort of paediatric brain cancers and identified a counter-correlation between telomerase expression and the genes of the pre-replicative complex. Moreover, the analysis of ALT markers in a group of 20 patients confirmed the association between ALT and increased RPA and decreased H3K9_me3_ localization at telomeres. *Conclusions*: Our study suggests that telomere maintenance mechanisms may act as a driver of telomeric DNA replication and chromatin status in brain cancers and identifies markers of ALT that could be exploited for precise prognostic and therapeutic purposes.

## 1. Introduction

ALT, an alternative mechanism to maintain telomere length based on homologous recombination, is found mostly in tumors with a mesenchymal origin (sarcomas) and in a subset of malignant pediatric brain tumors [1], including High Grade Glioma (HGG, 51%), Diffuse Intrinsic Pontine Glioma (DIPG) (18%), Choroid Plexus Carcinoma (CPC) (22.6%), and Primitive Neuroectodermal Tumors (PNET) (11.6%) [2,3]. In contrast with adult glioblastoma, mutations within the promoter region of the telomerase catalytic subunit, *TERT*, leading to its overexpression, occur at a lower rate in pediatric tumors (3–11% compared to 55–83% in adult tumors) [4,5]. Indeed, pediatric brain tumors show distinctive genetic mutations, which suggest that the mechanisms of cancer development and progression are different from those identified in adult brain tumors, where ALT develops in approximately 15% of cases, and is associated with IDH1 mutations and better prognosis [6]. Exon sequencing of paediatric HGGs identified recurrent somatic mutations (K27M, G34R/V) in histone H3 [7,8] and inactivating mutations in the Death-domain associated protein/Alpha thalassemia-mental retardation (DAXX/ATRX) genes, leading to DNA hypomethylation [9,10,11]. These findings suggest that telomeric chromatin plays an important role in ALT. Altered histone modifications in subtelomeric regions in association with ATRX loss result in deregulated telomere length and chromosomal instability, features that are often associated with ALT [12]. 

The World Health Organization (WHO) describes more than 100 categories and subtypes of primary juvenile brain tumours, according to their presumed cell of origin and histological classification [3]. The differences with the adult brain tumors, the intratumor heterogeneity and the young age of the patients make it hard to treat these tumors; this is mainly due to limited molecular markers that can be linked to response to treatment. In recent years, the discovery of unique biological drivers linked to ALT suggested promising strategies for the development of new powerful therapies. Recently, Next Generation genomic platforms identified common molecular features shared by different types of paediatric brain tumours [2,8,12]. Since 2016, the updated classification uses molecular parameters to define many tumour entities [13]. However, the study of TMMs adopted by different paediatric tumors remains challenging based on genomic, epigenetic and expression data. More accuracy on TMMs of paediatric brain cancer can be obtained by careful analysis of pathological specimens, including the combined staining for ALT-associated PML bodies (APB) [14] and TERT, but these approaches are still not enough as stainings are variable and their diagnostic value is unclear. More reliable for the diagnosis of ALT would be quantitative detection of DNA C-circles and telomere content [15]; however, a systematic analysis of these parameters in pathological specimens is not yet routinely performed; in addition, no treatments have been developed or proposed for ALT vs. telomerase positive tumors.

In an attempt to identify drivers and/or markers of ALT in pediatric brain cancers, we made use of preclinical models of the disease. In our lab, we have developed a juvenile model of brain tumour by overexpression of oncogenic RAS in zebrafish neural progenitor cells [16]. Although the zebrafish model is clearly different in many aspects from human, zebrafish telomeres (15–20 kb) are more similar to human telomeres (10–15 kb) than mouse telomeres (50–100 kb), and, although telomerase is constitutively active in some organs, the expression of Tert-mRNA, the catalytic subunit of telomerase, telomerase activity and telomere length decrease drastically with age, similarly to human tissue [17,18,19]. Zebrafish tumor models therefore could be very valuable to study telomere maintenance mechanisms. 

In this model, brain tumor cells use ALT to maintain their telomeres, but re-expression of *tert* in the same tumors prevents development of ALT and prolongs survival [20]. Here we compared gene expression between the two models. Comparison between telomerase positive and ALT zebrafish brain tumors identifies increased expression of genes of the pre-replicative complex as hallmarks of ALT. We also used these observations to interrogate available data from the TCGA consortium and validate them in a cohort of human paediatric brain tumors, thus establishing a protocol for the detection of ALT/telomerase status in pathological specimens.

## 2. Results

### 2.1. The Genes Involved in the Activation of the Pre-Replicative Complex May Play a Role in the Switch between ALT and Telomerase-Dependent TMM in Brain Tumors

**Figure 1:**
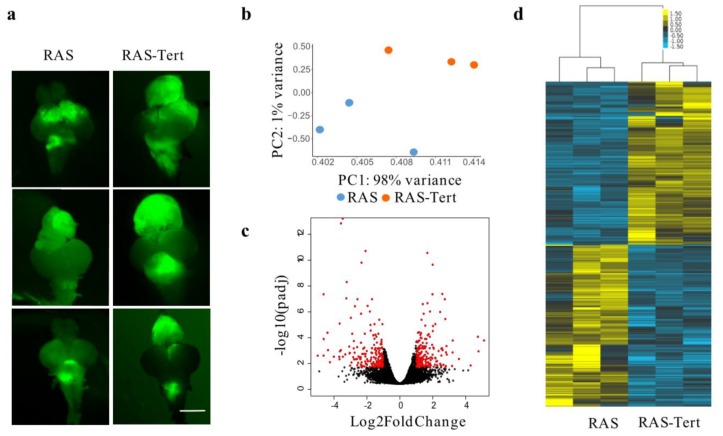

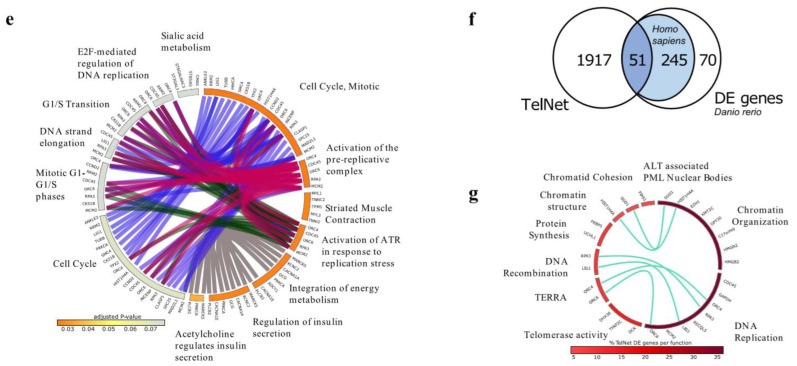
Analysis of RNA-Seq showed a class of genes altered in zebrafish brain tumors with different TMMs. (**a**) Images showing zebrafish RAS and RAS-Tert brains used for the RNA-Seq analysis. The expression of eGFP-HRASV^12^ was induced in a population of brain progenitor cells using the driver line zic:GAL4 [16]. The tumour masses in 1-month old fish are visualised through eGFP expression. Scale bar: 0.5 mm. (**b**) Principal component analysis (PCA) of the gene expression counts (Trimmed Mean of M-values, TMM) showing the first versus the second principal component (PC). Samples in the two conditions are highlighted in different colors. (**c**) Volcano plot representing −log10 Benjamini-Hochberg (BH) adjusted *p*-value and log2 Fold Change of genes in the comparison RAS and RAS-TERT. In red differentially expressed genes with adjusted *p*-value < 0.05 and log2 Fold Change >1 or <−1 are highlighted. (**d**) Heatmap showing the 366 differentially expressed genes between brain tumors from RAS and RAS-Tert, hierarchically clustered with average linkage and uncentered correlation metric with cluster3, and displayed with tree view. (**e**) Circular bar representing Reactome pathways enrichment for the DE gene set, colored according to adjusted *p*-value, with the most significant in orange, drawn with circos. DE genes in the pathways are indicated on the bars and are connected to the same genes in other pathways. (**f**) Venn diagram showing the number of differentially expressed genes between RAS and RAS-Tert brain tumors, which have a human ortholog and are present in TelNet database. (**g**) Circular plot depicting TelNet functions of the differentially expressed genes reported in TelNet database and their connections.

We previously established two isogenic models of juvenile brain tumors in zebrafish, that differs for the TMM adopted by cancer cells [20]. The two models are based on the somatic expression of the human oncogene HRASV^12^ in neural progenitor cells; the first model (called RAS) (Figure 1a, RAS) resembles the molecular mesenchymal subtype of glioblastoma [16] and uses ALT for telomere maintenance [20]. 

By contrast, in the second model (called RAS-Tert) (Figure 1a, RAS-Tert), the re-expression of the two components of telomerase, under the same promoter that drives oncogene expression, prevents ALT and lead to extended survival [20]. To identify molecular drivers and /or markers of ALT, we performed transcriptome analysis through RNA-sequencing (RNAseq) in three ALT and three telomerase positive (+) tumors (Figure 1a). Principal component analysis (PCA) identified two well separate clusters corresponding to ALT and telomerase+ brain tumors (Figure 1b). By comparing gene expression among the two different tumor types, we identified a limited set of genes that were significantly deregulated (Figure 1c, red dots).

The analysis of differentially expressed (DE) genes using DESeq2, showed 366 DE genes (Figure 1d, see Table S1). To investigate the biological pathways altered between ALT and Telomerase+ brain tumors, we first identified the human orthologs of the zebrafish DE genes (RAS-Tert vs. RAS) using Biomart and Beagle and then performed pathways enrichment analysis using Reactome with the significantly enriched pathways having adjusted *p*-value (padj) < 0.1 (see Table S2). We found that eight out of thirteen enriched pathways were strictly related to cell cycle and DNA replication (Figure 1e, see Table S2). The most significant pathways included “Cell cycle–mitotic” and “Activation of the pre-replicative complex” (padj < 0.05) (Figure 1e, see Table S2). 

We then evaluated whether the 366 DE gene cohort was enriched for genes known to regulate telomere biology. For this purpose, we used the Telnet database, which contains a list of genes that have been reported to be involved in telomere maintenance mechanisms [21]. We established that the 17.3% of DE genes (51/296 DE genes with human orthologous) were listed in Telnet database (Figure 1f); we used the information provided by TelNet on gene-specific functions to classify the DE genes identified in our brain tumor models. We found that the most significant DE genes belonged to the category: DNA replication and chromatin organization, according to the Telnet database (Figure 1g and see Table S3).

To strengthen the correlation between the higher expression of genes of the pre-replication complex and ALT, we analysed the expression of the most significantly affected genes, *orc4, orc6, mcm2 and rpa3*, in two additional ALT models. The first model was a human cell line (HeLa, telomerase+) which, when depleted of the histone chaperon ASF1, develop ALT [22], and the second model was the larval zebrafish brain tumor model, before and after ALT development (which occurs around 21 dpf, [20]). In both models we found a significant increase in the expression of the above genes upon ALT development (Figure S1).

Thus, Reactome pathway enrichment analysis, Telnet classification and experimental evidence in two additional models suggest that DE genes regulating DNA replication processes, especially the activation of the pre-replicative complex, may be important markers of the switch towards ALT in zebrafish brain tumors. Therefore, we selected this pathway for further analysis of human data. 

### 2.2. Correlation between Expression of Genes of the Pre-Replicative Complex and TERT Levels

**Figure 2:**
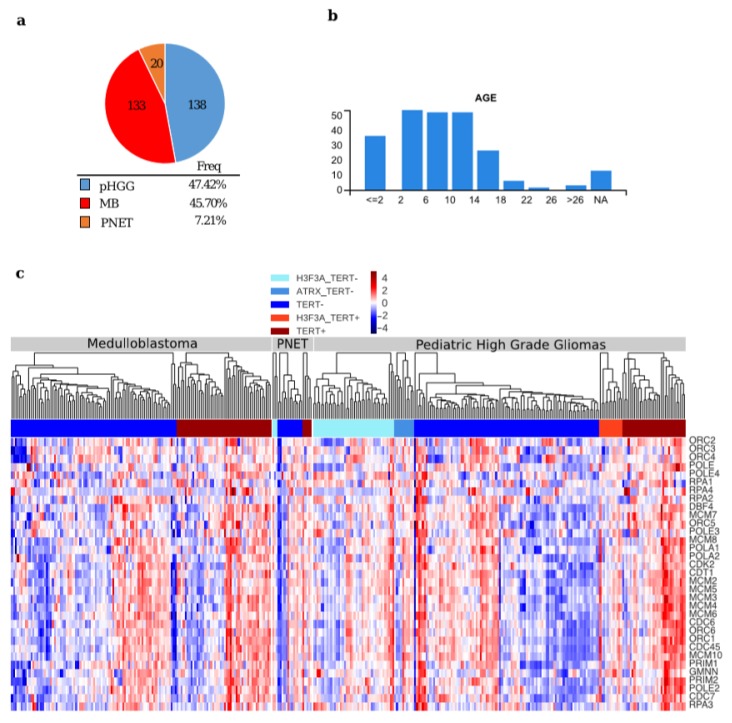
General features in paediatric brain tumor samples used for comparison (**a**) Pie chart depicting the tumor samples from the pediatric cBio Portal used in the analysis; (**b**) Diagrams showing the different ages of the patients from the pediatric cBio Portal; (**c**) Hierarchical clustering of the expression values of the genes of the Reactome pathway: “Activation of the pre-replicative complex” (Homo sapiens R-HSA-68962) in samples from the paediatric cBio Portal. The samples were divided per tumor type, according to the presence of mutations in H3F3A and/or ATRX, and TERT expression level (TERT+: Z-score > 0, TERT−: Z-score < 0).

We analyzed the expression of the 33 genes reported by Reactome (Homo sapiens, R-HAS-68962) as composing the ”Activation of the pre-replicative complex” pathway (for list of the genes see Table S4) in TCGA expression profiles of different paediatric/juvenile brain tumors (https://pedcbioportal.org/), including 133 medulloblastomas (MB), 20 primitive neuroectodermal tumors (PNET) and 138 paediatric high grade glioma (HGG) (Figure 2a–c). Moreover, we correlated the expression of the 33 genes involved in the activation of the pre-replicative complex pathway with TERT expression levels. We also reported the presence of somatic mutations in the genes encoding H3.3 and ATRX, as these mutations are frequently found in ALT paediatric brain tumors [7,9] (Figure 2c). 

**Figure 3:**
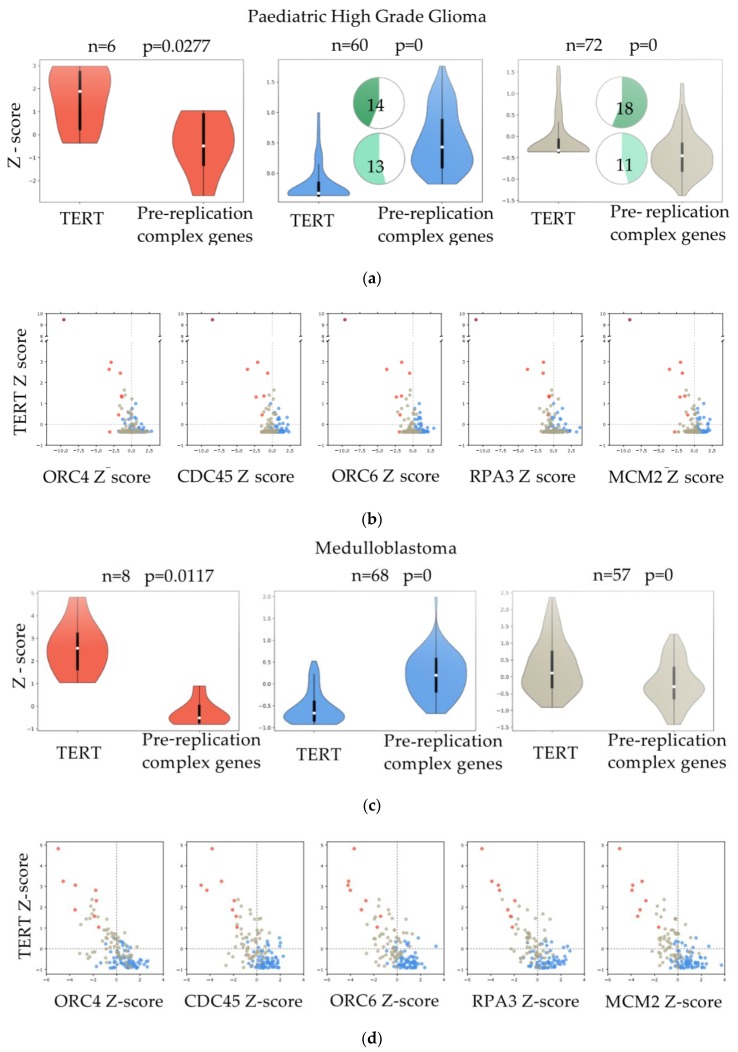

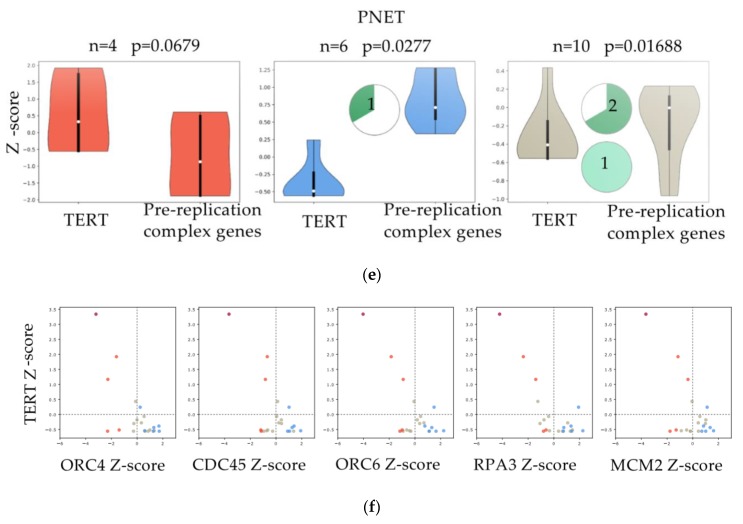
Correlation between the expression of genes of the pre-replicative complex and telomerase levels. (**a**,**c**,**e**) Violin plots comparing the expression of TERT and the mean expression of genes in the: “Activation of the pre-replicative complex” pathway in paediatric High Grade Glioma (pHGG), Medulloblastoma and PNET. Samples were divided into three groups by K-means clustering. Pie chart plots show the percentage of samples (actual numbers are indicated) with H3F3A (dark green) or ATRX (light green) mutations for each of the three groups. No mutational data were reported for MB. (**b**,**d**,**f**) Scatter plots of pHGG, Medulloblastoma and PNET showing the expression of TERT (y-axis, in Z-score) vs. the expression of five genes (ORC4, CDC45, ORC6, RPA3, and MCM2) in the: “Activation of the pre-replicative complex” pathway (x-axis, in Z-score). Samples marked in different colors belong to the three different groups as clustered by K-means.

The hierarchical clustering on each tumor type (Figure 2c) suggests that all the genes of the pathway work as a module, when the pathway is activated, all the genes are upregulated and vice-versa, leading to the identification of different segregation groups. To better understand how the pathway is activated in relation to low TERT expression, we clustered the data using K-Means (Figure 3a,c,e). We found three different groups, and the behaviour of TERT was anti-correlated with the mean expression of the pre-replicative complex genes in two out of three k-groups (Figure 3a,c,e).

Mutations in H3F3A (pHGG *n* = 32; PNET *n* = 3) and ATRX (pHGG *n* = 24, PNET *n* = 1) reported for some samples were clustered in group 2 (low expression of TERT, high expression of the genes of the pathway) and group 3 (TERT Z-score close to 0, pathway Z-score close to 0) (Figure 3a,e). The profile of the five genes (ORC4, CDC45, ORC6, RPA3 and MCM2) found deregulated in zebrafish RAS-TERT vs. RAS tumors confirmed the presence of three different types of relationships: some tumors showed that when TERT was up-regulated (Figure 3b,d,f, Y-axis Z-score > 0) the five genes of the pre-replicative complex were down-regulated (x-axis Z-score < 0) and vice-versa; the third group of samples showed that when the levels of TERT were close to zero, also the five genes of the pathway were close to zero (Figure 3b,d,f). 

Thus, both in zebrafish brain tumor models and in human juvenile brain tumors, the expression of TERT is mostly anti-correlated with genes of the pre-replication complex, suggesting that in ALT tumors more active DNA replication may contribute to telomeric dysfunction.

### 2.3. Identification of TMMs in a Panel of Twenty Human Juvenile Brain Tumors

**Figure 4:**
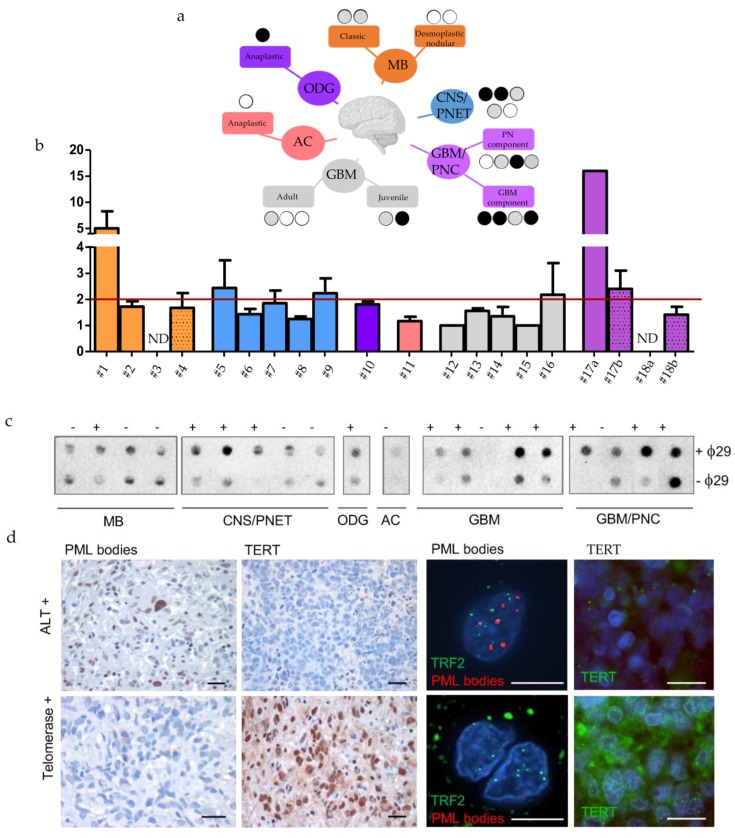
Identification of TMMs in a cohort of twenty human paediatric brain tumour. (**a**) Tree representation of the human cases analysed here and the TMMs identified. Black, grey and white circles indicates the results of CCA analysis (black: positive at 2 tests, grey: positive at 1 test and white: negative). (**b**) Identification of human brain tumors positive or negative for C-Circles, analysed by qPCR (Bars represent mean of fold changes +/- s.e.m of two independent experiments) and (**c**) dot blot. The red line shows the limit above which ALT is detected. MB = Medulloblastoma, CNS/PNET= Central Nervous System Primitive NeuroEctodermal Tumors, ODG = oligodendroglioma, AC = astrocytoma, GBM = glioblastoma, GBM-PNC = Glioblastoma with Primitive Neuronal Component. (**d**) Immunohistochemical and immunofluorescent stainings of PML bodies, TERT and PML bodies/ TRF2 in two representative cases of juvenile brain tumors, ALT or telomerase+, as indicated. Calibration bars: 10 µm.

The analysis reported above found an anti-correlation between TERT expression and the levels of five genes of the pre-replicative complex, but could not assign an ALT status based on gene expression data. To search for a definitive correlation between ALT and DNA replication in human brain tumors, we evaluated TMMs in a panel of 20 primary, mostly paediatric and juvenile, brain tumors of different histology. We performed the TMM characterization in paraffin-embedded brain tumors, distributed as follows: four MB, five Central Nervous System Primitive NeuroEctodermal Tumors (CNS-PNET), one oligodendroglioma (ODG), one astrocytoma (AC), two juvenile glioblastomas (GBMs) and four rare histological variants of conventional GBM with Primitive Neuronal Component (GBM-PNC); three adult GBMs were added as controls (Figure 4a, see Table 1). First, we examined the presence of C-Circles using Q-PCR and dot blot analysis in the tumors (Figure 4b,c, see Table 1). Using two methods for detecting C-Circles (dot blot relative intensity, see Table S6, and telomeric qPCR) allowed us to identify seven cases, positive to both tests for ALT including, two CNS-PNET, 1 ODG, one juvenile GBM and three GBM/PNC (see Table 1 and black dots in Figure 4a). We also evaluated the presence of PML bodies and the expression of TERT by immunohistochemistry (Figure 4d); although the levels of TERT may not be directly related to telomerase activity, low or absent TERT expression could be an additional evidence supporting the activation of ALT in the positive cases. The seven C-Circle positive cases, were mostly positive for PML foci, co-localized with TRF2 (Figure 4d, upper panel; see Table 1); some were negative for TERT (three out of seven, Figure 4d, green highlight in Table 1) suggesting that ALT is the main TMM active in these tumors; three C-Circle/PML positive tumors, including one juvenile GBM and two GBM/PNC were also positive for TERT expression (pink highlight in Table 1). Five tumors negative for C-Circles and PML bodies (blue highlight in Table 1) resulted positive for TERT (Figure 4d), suggesting that telomerase is the most representative TMM in these tumors. For some cases, it was difficult to attribute a clear TMM classification. These included three CNS-PNET and three MB tumors, which showed low score for PML bodies and no signal for TERT and C-Circle, whereas two out of four GBM-PNC and 1 AC resulted almost negative for all three markers (see Table 1, no highlights).

A surprising result was relative to GBM/PNC tumors, where the presence of C-Circle, PML bodies and TERT was confined mostly to the GBM component, whereas the PNC component showed an absence of TERT expression and no PML bodies immunoreactivity (pink highlight in Table 1). The molecular profile showed that only one case of the seven ALT tumors presented the IDH1^R132^ mutation and the loss of ATRX (no. 17 in Table 1). A positive C-Circle signal is considered a reliable indicator of ALT [15]. However, single-stranded C-Circles could be lost in paraffin-embedded material during sample preparation; for this reason, we used additional parameters to classify tumors as ALT, including, abundance of PML bodies, absence of detectable TERT expression and the available data regarding the molecular profile (Table 1).

Using this combination of markers, the analysis allowed us to identify: A set of juvenile brain tumor which were clearly ALT (see Table 1, green highlight), a set of brain tumors which appeared telomerase-dependent (see Table 1, light blue highlight), a set of ALT/TERT+ tumors (see Table 1, pink highlight), and a set of tumors with uncharacterized TMM mechanisms. The tumors with identified TMMs were then used for the analyses showed in the next set of experiments, aimed at localizing markers of replication/heterochromatin dysfunctions at telomeres of ALT or telomerase+ tumors. In conclusion, ALT features frequently occur in different types of juvenile brain tumors, and the TMM status can be inferred from conventional pathology material.

### 2.4. The Reactivation of Telomerase Rescues ALT by Preventing Replication Stress and by Reorganizing Telomeric Chromatin

**Figure 5:**
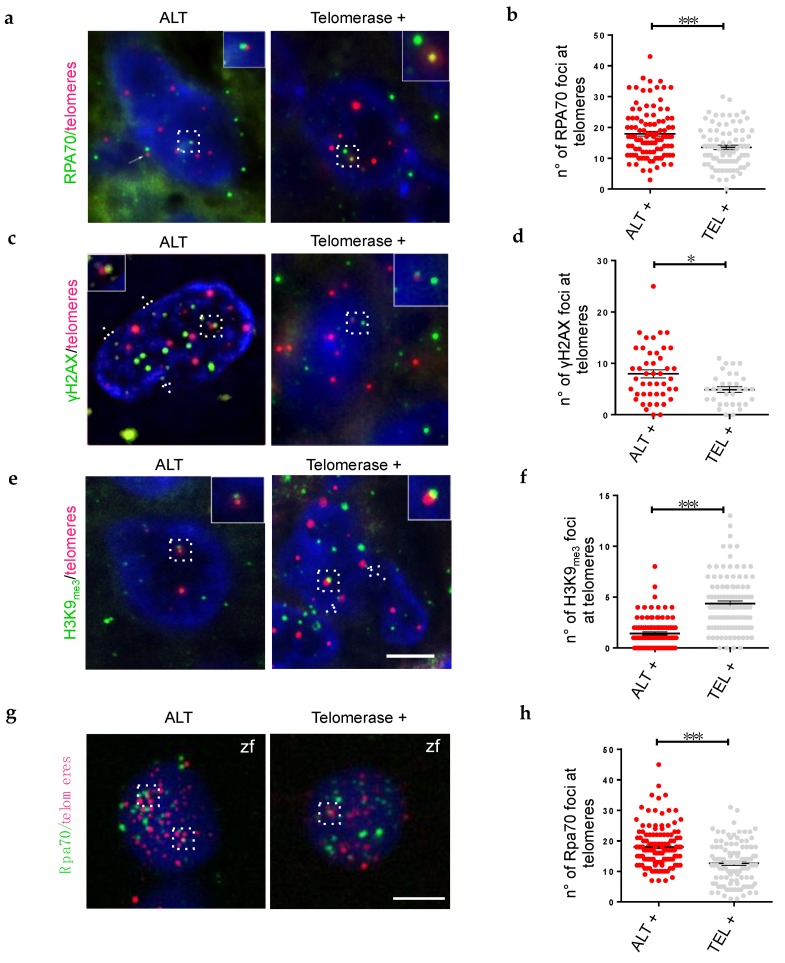
The presence of telomerase leads to a reorganization of telomeric chromatin, decrease DDR and replication stress. (**a**,**c**,**e**) Fluorescent microscope images of a single plane of focus of representative ALT, and telomerase+ human brain tumor cells, stained by immunofluorescence (green) combined with Q-FISH (magenta). Antibody against replication stress marker (a, RPA70), DNA damage marker (c, γH2AX), and chromatin methylation marks (e, H3K9me3) were used and counterstaining with DAPI. Insets, dashed frames and arrows show co-localization of immunofluorescence and Q-FISH spots. Scale bar: 5 µm. (**b**,**d**,**f**) Immunofluorescence quantification expressed as the number of foci per nucleus that colocalized with telomeres (*n* = 25–60 nuclei) for the corresponding samples in (**a**,**c**,**e**); * *p* < 0.05, *** *p* < 0.001 between the indicated groups. (**g**) Fluorescent microscope images of representative ALT and telomerase+ zebrafish brain tumor cells, stained by immunofluorescence (green) combined with Q-FISH (magenta). Antibody against a replication stress marker (RPA70), were used and counterstained with DAPI. Scale bar: 5 µm. (**h**) Immunofluorescence quantification expressed as the number of foci that colocalized with telomeres per nucleus (*n* = 25–60 nuclei) for the corresponding samples in (**g**); * *p* < 0.05, *** *p* < 0.001 between the indicated groups.

Our analysis of zebrafish brain tumors revealed a reduced expression of genes of the pre-replicative complex and an increased expression of genes driving heterochromatin deposition as hallmarks of the telomerase rescue. For this reason, we decided to search for signs of replication stress/stalled replication forks (RPA complexes), associated to increased double-strand breaks (γH2AX foci), and assessed the status of heterochromatin (H3K9_me3_ foci) specifically at telomeres of ALT or telomerase+ human brain tumors. In both zebrafish and human tumors, we found a significant increase of telomeric RPA+ foci in ALT brain tumor cells compared to telomerase+ tumors (Figure 5a,b,g,h).

Next, we investigated the presence of telomere dysfunction-induced foci (TIF, an indication of DNA damage) by immunostaining for γH2AX. The number and localization of DNA damage foci at telomeres of ALT tumors were significantly higher than in telomerase+ tumors (Figure 5c,d). This result demonstrates that in ALT paediatric brain tumors the levels of DNA damage at telomeres is higher than in telomerase+ brain tumors, suggesting that telomerase can help resolving the replication stress, and reduce DNA damage in telomeric regions. Finally, we investigated the distribution of heterochromatin foci at telomeres, using H3K9_me3_ as marker. We found a dramatic decrease in telomeric chromatin histone 3 lysine 9 methylation in ALT brain tumors (Figure 5e,f), similar to what we described in zebrafish brain tumors [20]. 

Thus, quantification of RPA, γH2AX and H3K9_me3_ foci localized at telomeres in ALT or telomerase+ brain tumors suggests that the rescue of ALT by telomerase overexpression may be mediated by a reduction of telomeric DNA replication, with consequently reduced replication stress, and re-establishment of protective telomeric heterochromatin, which also leads to a reduction of TIFs. As the effects of telomerase overexpression at the molecular level involve a reduction in the expression of genes of the pre-replicative complex, and a consolidation of genes involved in heterochromatin maintenance, it is tempting to speculate that telomere maintenance mechanisms may coordinate DNA replication and chromatin status at telomeres in brain cancers.

## 3. Discussion

ALT is thought to be triggered by factors that alter chromatin structure at telomeres; these include reduced deposition of histone 3 variants, due to defective histone chaperon ATRX activity or direct histone 3 mutations, altering H3K27 methylation, and heterochromatin formation. Nevertheless, the epigenetic nature of human telomeres remains controversial and while a number of studies have shown ALT telomeres to be in a less compact state than telomerase-positive telomeres [23,24], deposition of heterochromatic levels of H3K9_me3_ at ALT telomeres have also been recently described [25,26,27]. But how altered chromatin organization triggers ALT and whether the choice of TMM is a cause or a consequence of the telomeric chromatin status in cancer is not fully understood. 

Here we used two isogenic models of juvenile brain tumors in zebrafish, based on the overexpression of RAS in neural progenitor cells; although ALT paediatric brain cancers are often related to mutations in histone genes, and/or in genes related to chromatin remodelling, we found that RAS-induced juvenile fish brain tumors develop ALT as main TMM. In view of the prevalence of mutations causing activation of MAP kinase and phosphoinositide 3-kinase (PI3K) signalling in more than two-thirds of high-grade pediatric gliomas [28], the occurrence of ALT in this model may be very relevant to pediatric/juvenile brain tumors.

By investigating the RAS zebrafish cancer model we found that ALT can develop not only as a consequence of one of the mutations that alter chromatin structure at telomeric regions, but as a direct consequence of the lack of telomerase expression. Indeed, sustained expression of tert in the neural progenitors that initiate tumorigenesis prevented ALT development [20]. Previous evidence in human cancer cell lines have shown that expression of hTERT does not abolish ALT once ALT is established [29]. However, genetic ablation of telomerase in a mouse cancer model can lead to ALT development [11], suggesting that telomerase activity can prevent the emergence of ALT in cancer. In the zebrafish brain cancer model, sustained telomerase expression in tumor initiating cells not only prevents ALT, but also re-establishes the heterochromatin status at telomeres, reduces DNA replication stress and represses TERRA transcription [20]. Previous studies have indicated the shelterin complex as instrumental in preventing DNA replication stress and DNA damage response signaling through recruitment of helicase enzymes to telomeres [30,31,32] or formation of the T-loop structure [33,34]. The shelterin proteins have also been proposed to regulate telomeric chromatin compaction [35]. However, two recent studies reported that DDR at telomeres which were depleted of TRF1 and TRF2 occurs in the absence of chromatin decompaction [36,37]. Thus, DDR localization at telomeres may be dependent on telomeric heterochromatin structure, including posttranslational modifications [38], rather than chromatin compaction. On the basis of our observations, it would be interesting to investigate whether telomerase activity at telomeres can act synergistically with the shelterin complex in preventing/releaving replication stress at telomeres and participate in the heterochromatin organization of chromosome ends. 

What is the role of telomeric DNA replication in ALT+ cells? Our study on DE genes between ALT and telomerase+ brain tumors indicates that the pre-replicative complex may play a role or be an important marker in ALT development or maintenance. The pre-replicative complex involves the ordered assembly of a number of replication factors including ORCs, CDC6p, CDT1p, and MCM2–7 on the two sides of the origin of replication. Further assembly leads to the recruitment of the mature replisome, which is composed by CDC45, GINS, and POLε, and involves DDK and CDK kinase activities to complete assembly and prime the complex for helicase activation that is accomplished by MCM10 and RPA [39]. Thus, key factors contributing to different steps in the assembly and function of the pre-replicative complex are increased in expression in ALT brain tumors, including the small RPA subunit, RPA3, which, intriguingly, has been also described as part of the Ctc1-Stn1-Ten1 complex, which binds to single-stranded DNA and protects telomeres, independently of the shelterin complex [40]. 

Expression of the genes of the pre-replicative complex is anti-correlated with *TERT* expression both in human pediatric and in zebrafish juvenile brain tumors. Kurth and Gautier [41], have shown that pre-replicative complexes assemble within telomeric DNA and can be converted into functional origins. Indeed, the majority of telomeric DNA is duplicated by conventional DNA replication, but telomerase may control the amount of DNA replication at telomeres by binding and sequestering TRF2, which has been shown to recruit origin of replication complexes (ORCs) through its TRFH dimerization domain [42]. Thus, in the absence of telomerase, an excess of ORC and TRF2 contribute to increase telomeric DNA replication, with the excess telomeric DNA being used for recombination and for ALT associated C-Circles. 

In ALT cells increase of DNA replication is linked to decrease heterochromatin formation. The open chromatin status may lead to the incorporation of non canonical variant repeats, which alter the binding of the shelterin complex, thus reinforcing the loop between DNA-replication and telomere de-protection. In addition, disruption of telomeric chromatin environment results in higher levels of TERRA transcription. TERRA transcripts may participate in ALT induction by multiple mechanisms: through formation of DNA:RNA hybrids, or R-loops, which may promote homologous recombination among telomeres [43]; by interfering with ATRX functions, as described in mouse embryonic stem cells [44]; by impacting replication of telomeric DNA [45]. In the ALT zebrafish brain tumor model we detected high levels of Terra expression [20].

The mechanisms by which telomeric chromatin participates to the specific TMMs remain to be elucidated. Interestingly, abrogation of DNA methyltransferases 1 (DNMT1) or DNMT3A and DNMT3B [46] resulted in telomere elongation, increased telomeric recombination and ALT development, in mouse embryonic stem cells. These findings suggest that DNA methylation prevents ALT development. On the other hand, Gauchier et al. [47] recently reported that telomeric H3K9 trimethylation performed by the histone methyltransferase SETB1, which associates to telomeres, promotes heterochromatin formation at telomeres. Excessive heterochromatin formation by SETB1 in mouse embryonic stem cells leads to the emergence of ALT features while loss of SETB1 and consequent reduction of the heterochromatic mark H3K9_me3_ at telomeres resulted in altered telomere protein composition with reduced binding of heterochromatin proteins 1 (HP1α, HP1β and HP1γ), DNMT3A, DNMT3B and DNMT3L and suppresses ALT. Heterochromatin is a feature of telomeric repeats and it is known to spread to subtelomeric regions in all species, maintaining a “silent” chromatin environment, but even more importantly, contributing to the 3D organization of the genome of differentiated cells. Cancer is challenging this organization, not only because it imposes DNA replication at increased rates, but also because it is characterized by decreased DNA methylation and euchromatinization [48]. These changes involve telomeric chromatin, which must be protected, because of its fundamental role in 3D genome organization [49]; telomerase which is frequently re-expressed in cancer may actively participate in these mechanisms. The non canonical role of telomerase, hypothesized by Chan and Blackburn [50], may well include organization and maintenance of heterochromatin, regulation of DNA replication in the telomeric region, and perhaps resolution of DNA damage resulting from stalled replication forks; in this regard, it has recently been shown that, in human (as in yeast), telomerase recruitment to telomeres, besides being regulated by the shelterin complex [51] is also driven by the DNA damage sensor kinases, ATM and ATR [52].

## 4. Materials and Methods 

### 4.1. Maintenance of Zebrafish Lines

Adult zebrafish (*Danio rerio*) were housed in the Model Organism Facility—Center for Integrative Biology (CIBIO) University of Trento and maintained under standard conditions [53]. All zebrafish studies were performed according to European and Italian law, D.Lgs. 26/2014, authorization 148/2018-PR to M. C. Mione. Fishes with somatic and germline oncogene expression were generated as described [16,20]. The following zebrafish transgenic lines were used in the course of this study: *Et(zic4:Gal4TA4, UAS:mCherry)_hzm5_* crossed or not to *Tg(UAS:eGFP-HRAS_G12V)_io006_*_._

### 4.2. Human Pathology Material

Human brain tumor samples were retrieved from the archive of Department of Pathology, Spedali Civili di Brescia, University of Brescia, in agreement with protocols approved by the Institutional Review Board. Specifically, for the retrospective and exclusively observational study on archival material obtained for diagnostic purpose, patient consent was not needed (Delibera del Garante n. 52 del 24/7/2008 and DL 193/2003).

### 4.3. C-Circles Assay

C-Circles assay was performed as described [22], following genomic DNA extraction from paraffin sections with the Quick-DNA FFPE Kit (Aurogene, Rome, Italy). Briefly 30 ng of genomic DNA was combined with 0.2 mg/mL BSA, 0.1% (v/v) Tween 20, 1 mM each dATP, dTTP, dGTP, 4 µM dithiothreitol (DTT), 1× Φ29 DNA polymerase buffer, 7.5 U Φ29 (New England Biolabs, Pero, MI, Italy). Rolling circle amplification (RCA) reactions were performed by incubation at 30 °C for 8 h, plus 20 min at 65 °C. Reactions without the addition of Φ29 polymerase were included as a control (“−Φ29”). For dot blot detections, the CCA products (plus 40 μL 2× SSC) were dot-blotted onto 2× SSC-soaked positive nylon membrane, thanks to a 96-well Bio-Dot Microfiltration Apparatus (BioRad, Segrate (MI), Italy). The membrane was UV-crosslinked for 3 min/each side and hybridized with probe (CCCTAA)_8_ labelled with DIG Oligonucleotide 3’-End labeling Kit (Sigma-Aldrich, Milano, Italy) and developed as described [54]. Image Lab™ Software (BioRad) was used to analyse dot intensity. The result of the C-Circle assay dot blot was evaluated according to [15]. q-PCR detection was performed as described [22]. Briefly, CCA products were diluted 4 times in water and used as templates in a qPCR reaction using telo-Forward (300 nM) 5’-CGGTTTGTTTGGGTTTGGGTTTGGGTTTGGGTTT GGGTT-3’ and telo-Reverse (400 nM) 5’-GGCTTGCCTTACCCTTACCCTTACCCTTACCCTTA CCCT-3’ primers. qPCRs using 36B4 primers Forward (300 nM): 5’-CAGCAAGTGGGAAG GTGTAATCC-3’ and Reverse (500 nM): 5’-CCCATTCTATCATCAACGGGTACAA-3’ were performed for Single Copy Gene (SCG) normalization. All qPCRs were done in triplicate, a positive control U2OS (ALT +) and no DNA controls were included in each PCR run. Each telomere Ct was normalized with the SCG Ct (normTEL). The CC abundance of a sample was calculated as (normTEL in +Φ29) − (normTEL in −Φ29). ALT activity was considered significant if at least twice than the levels without Φ29 polymerase.

### 4.4. Immunostaining on Paraffin-Embedded Sections

Briefly, 2-μm-thick paraffin sections were deparaffinized and rehydrated. Endogenous peroxidase activity was blocked with 0.3% H_2_O_2_ in methanol for 20 min. Antigen retrieval (when necessary) was performed in either 1.0 mM EDTA buffer (pH 8.0) or 1 mM Citrate buffer (pH 6.0). Sections were then washed in TBS (pH 7.4) and incubated primary antibodies diluted in TBS 1% BSA at room temperature for 1 h. The reaction was revealed by using Novolink Dako EnVision+Dual Link System Peroxidase (Dako Cytomation, Glostrup Denmark) followed by DAB and slides counterstained with hematoxylin. For double immunohistochemistry, after completing the first immune reaction using the anti-PML antibody, the anti-TRF-2 was revealed by Mach 4 Universal AP Polymer kit (Biocare Medical, Pacheco, CA, USA) using Ferangi Blue (Biocare Medical) as chromogen and nuclei were counterstained with Methyl Green. For immunofluorescence, secondary antibodies conjugated with FITC and/or Texas Red was used and nuclei were counterstained with DAPI. The antibody used and their dilutions were as follows: PML bodies 1:100 (Santa Cruz Biotechnology, Dalla, TX, USA); TERT 1:100 (Novusbio, used for immunohistochemistry, Centennial, CO, USA); TERT 1:100 (Abcam, used for immunofluorescence, Cambridge, UK); TRF-2 1:200 (Novusbio). Images were acquired through an Olympus DP70 camera mounted on an Olympus Bx60 microscope using CellF imaging software (Soft Imaging System GmbH, Munster, Germany).

### 4.5. Immunofluorescence Combined with Q-FISH

Cell suspensions derived from zebrafish brain tumors were seeded on polylysine (1 µg/mL) (Sigma-Aldrich, Milano, Italy) slides, whereas paraffin sections from human brain tumor were deparaffinized and rehydrated as described above before processing for immune-fluorescence. Briefly, after 1 wash in TBS 1× for 5 min, slides were fixed in 2% paraformaldehyde containing 2% sucrose for 10 min at RT and then washed twice in TBS, followed by permeabilization with 0.5% Triton for 15 min. After 3 washes in TBS, slides were incubated 1 h at RT in blocking buffer (5% Normal Goat Serum (NGS), 0.1% Triton for H3K9_me3_ or 0.5% BSA, 0.2% Gelatin cold water fish skin in 1× PBS) and then overnight at 4 °C with primary antibody with the following dilutions: RPA70 1:200 (Thermo Fisher, Waltham, MS, USA), γH2AX 1:300 (Merck-Millipore, Milano, Italy), H3K9_me3_ 1:500 (Abcam). After three washes in blocking buffer, slides were incubated with secondary antibody (goat-anti-mouse 488 or Goat-anti-rabbit 488 Thermo Fisher) 1:500 for 2h at RT. After incubation, slides were washed 3 times in 1× TBS (5 minute each) and 1 time in Q-FISH washing buffer (0.1% BSA, 70% formamide, 10 mM Tris pH 7.2). Then FISH was performed with PNA TelC-Cy3 probe (PANAGENE, Yuseong-gu, Daejeon, Korea) as described [20]. Nuclei were counterstained with DAPI. Z-stacks Images were captured at 100× magnification (Plan Apochromatic 100×/1.45 oil immersion objective) using an inverted Ti2 fluorescent microscope (Nikon, Tokyo, Japan) equipped with a monochromatic Andor Zyla PLUS 4.2 Megapixel sCMOS camera (Oxford Instruments, UK). Images were processed for background subtraction using Fiji/ImageJ. Colocalization analysis was performed with DiAna/ImageJ [55], calculating co-localization between objects in 3D, after 3D spot segmentation.

### 4.6. RNA-Sequencing Analysis 

Demultiplexed raw reads (fastq) generated from the Illumina HiSeq were checked using FASTQC tool (version 0.11.3). All samples passed the quality standards. Then we aligned to the reference genome Danio rerio assembly GRCz11 using STAR [56] with recommended options and thresholds (version 2.5) HTSeq-count (version 0.9.1) [57] was used to generate raw gene counts. Counts normalization to Trimmed Mean of M-values (TMM) for visualization methods was performed by edgeR package (v.3.24.3) [58]. The differential expression analysis was performed using DESeq2 package (v.1.22.2) [59] and for significance testing, the Wald test was used. Genes were considered differentially expressed with adjusted *p*-value < 0.05 and a log2 fold change greater than 1 or smaller than −1. For statistical analyses, the adjusted *p*-values were generated via the Benjamini-Hochberg procedure. For visualization, differentially expressed genes (Trimmed Mean of M-values, TMM) were hierarchically clustered with average linkage and uncentered correlation metric with cluster3 [60] and displayed with treeview [61]. Human orthologs were identified through Beagle Database (accessed in January 2019, and BioMart) [62] for 295 of the 366 differentially expressed genes). Functional annotation on human orthologs of differentially expressed genes was carried out through enrichr web tool [63] (accessed in May 2019) on Reactome pathways. Pathways were represented using Circos plots using Circos package [64]. Connection curves between pathways represent the same gene is present in both pathways, they were manually drawn. Genes involved in telomere maintenance were obtained from TelNet database [21]. Expression data (Z-scores) for the Activation of the pre-replicative complex (Homo sapiens R-HSA-68962 ) Reactome pathway and TERT, were downloaded from the Paediatric cBio Portal [65] for samples whose mutations and RNA Seq V2 data were both available, in the following cancer types: medulloblastoma (MB), primitive neuroectodermal tumor (PNET), paediatric high grade glioma (pHGG). Samples per tumor type were divided in groups according to TERT expression level (TERT+: Z-score > 0, TERT−: Z-score < 0) and the mutations identified (H3F3A, ATRX), and hierarchical clustering with euclidean metric was performed on each group. Per tumor type, the downloaded Paediatric cBio Portal expression data were clustered using K-Means. TERT expression was subtracted from the mean expression of the pathway genes, for each sample. The number of groups (k) was determined using the elbow method. The difference between the expression of TERT and the mean expression of the pathway was tested with the Wilcoxon signed-rank test. Statistics. Except for data from RNA-Seq analysis, all the graph and the statistical analysis (Mann-Whitney-nonparametric test, no Gaussian distribution, two-tailed, interval of confidence: 95%) were generated and calculated using Prism GraphPad software version 5.0 (GraphPad, San Diego, CA, USA). Details regarding number of samples used and statistical analysis are provided in the figure legends.

### 4.7. siRNA Transfections and Treatments

For siRNA knockdown we used the protocol described by O’Sullivan [22]. Briefly, ~700,000 HeLa cells were seeded in 10 cm dishes containing 9 mL growth medium without antibiotics. 2 h later cells were transfected with 5 µL siRNA smartpools from Dharmacon (Thermo Fisher): ASF1a; CCCUGAAAUUCCGUAAGUAUU and ASF1b; CCCUUGAGUACCAUUGAUCUU. siRNAs and Dharmafect were diluted in OptiMEM (Thermo Fisher) to a working siRNA concentration of 50 nM. Transfection medium was replaced with complete culture media 24 h later and cells were harvested at 72 h post transfection.

### 4.8. Analysis of Gene Expression by qPCR

For zebrafish larval heads and human cells, total RNA was extracted with TRIzol reagent (Invitrogen). Total RNA was cleaned up using RNeasy Mini Kit (Qiagen, Milano, Italy) following the manufacturer’s instructions and treated twice with DNase I (1 unit/μg RNA, Qiagen). The RNA concentration was quantified using nanodrop2000 (Thermo Fisher) and VILO superscript KIT (Thermo Fisher) was used for First-strand cDNA synthesis according to the manufacturer’s protocol. qRT-PCR analysis was performed using qPCR BIO Sygreen Mix (Resnova-PCR Biosystem, Genzano, Italy) using a standard amplification protocol. The primers used are reported in Table S5. Data analysis was performed with Microsoft Excel and Graphpad Prism. In all cases, each qPCR was performed with triplicate samples and repeated with at least two independent samples. Data are expressed as fold changes compared to controls (2^−∆ ∆Cq).

## 5. Conclusions

In conclusion, an anti-correlation between genes involved in DNA replication and *TERT* expression was discovered in paediatric brain tumors and reinforced the significance of a similar correlation found in zebrafish models of juvenile brain cancer, which use ALT or telomerase as telomere maintenance mechanism. The possibility of relating these changes to ALT was further proved by specific and quantitative analysis of ALT features in a set of pathological specimens from diverse paediatric brain tumors. Thus our study identifies gene expression changes that suggest an involvement of the pre-replicative complex in ALT-related telomeric dysfunctions. In addition, these changes lead to specific combinations of telomeric markers (DNA damage, stalled replication forks and heterochromatin), which, together with markers of ALT (c-circles and increased telomere content), could be exploited for precise prognostic purposes, leading to a standardized procedure for assessing ALT in view of specific therapeutic approaches.

## Figures and Tables

**Table 1:** Identification of TMMs in a panel of twenty human brain tumours.

Histological Code	Code	Tumor Type	Additional Features	Age (y/o)	Ccircles Dotblot	Ccircles qPCR	TERT	PML Bodies	PML/ TRF2	ATRX
12913/02 A1	# 1	**MB, Classic**	-	3	-	+	0	1	-	+
15553.3/06 A1	# 2	**MB, Classic**	-	32	+	-	0	2	+	+
8151/00 A3	# 3	**MB, Desmoplastic/Nodular**	-	1	-	ND	1	0	-	+
15189/01 A6	# 4	**Medulloblastoma, Desmoplastic/Nodular**	-	4	-	-	0	1	ND	+
12296/07 1G (VI)	# 5	**CNS-PNET**	mostly undifferentiated	3	+	+	0	1	+	NE
8306.2/01 (PD)	# 6	**CNS-PNET**	mostly undifferentiated	ND	+	-	0	2	ND	NE
11106/01 (PD)	# 7	**CNS-PNET**	mostly undifferentiated	ND	+	+	0	1	+	NE
22994/08 1B (VI)	# 8	**CNS-PNET**	with areas of neuronal differentiation	ND	-	-	0	1	-	NE
18345/14 1A	# 9	**CNS-PNET**	with areas of neuronal differentiation	ND	-	+	0	1	ND	NE
18689.2/16 K1	# 10	**Anaplastic ODG**	-	44	+	+	0	2	ND	+
930.1/15 A6	# 11	**Anaplastic AC**	-	59	-	-	0	0	-	+
15152/11	# 12	**GBM**	-	61	+	-	1	0	ND	+
24181.1/16 K1	# 13	**GBM**	-	21	+	-	2	0	-	+
20424.1/16 A1	# 14	**GBM**	-	50	-	-	2	0	-	+
15998.1/16 A3	# 15	**GBM**	-	49	+	-	1	0	-	+
39060.2/16 A4	# 16	**GBM**	mostly undifferentiated	33	+	+	3	3	+	+
27931.1/16 A3	# 17a	**GBM-PNC, IDH1-R132H mutated**	glioblastoma component	27	++	++	2	2	+	-
A1	b		primitive neuronal component		-	+	0	0	-	-
32563.1/15 A6	# 18a	**GBM-PNC**	glioblastoma component	76	+	ND	2	1	ND	+
A5	b		primitive neuronal component		-	-	0	0	ND	+
36357/15 A2	# 19°	**GBM-PNC**	glioblastoma component	68	+	+	3	1	+	+
A2	b		primitive neuronal component		+	+	0	1	+	+
6622.2/15 A3	# 20a	**GBM-PNC**	glioblastoma component	50	+	-	1	1	-	+
A3	b		primitive neuronal component		+	-	0	0	-	+

The table reports features of the 20 brain tumors analysed for C-Circles (by dot blot and Q-PCR), TERT, PML bodies, PML/TRF2 colocalization (by immunofluorescence) and ATRX protein expression (by immunohistochemistry). For each tumor, the table reports the histological code, the internal number code (this paper), patients’ age and additional features, according to Louis et al., 2016. Scores reflect combined intensity and percent of stainings and are as follows: 0 = negative/very low; 1 = low; 2 = medium; 3 = high. Colors of highlights represent the TMM classification, and are as follow: Green: ALT; Pink: ALT with TERT expression; Blue: Telomerase+; not highlight means no TMM classification. Scores are reported in the legend. ND = not detected; NE = not evaluated. MB: Medulloblastoma, CNS-PNET: Central Nervous System Primitive NeuroEctodermal Tumors, ODG: oligodendroglioma, AC: astrocytoma, GBMs: glioblastoma, GBM-PNC: rare histological variants of conventional Glioblastoma with Primitive Neuronal Component. Degree of immunostaining according to Figure S2.

## Supplementary Materials

The following are available online at https://www.mdpi.com/2072-6694/12/4/1028/s1, Figure S1: qPCR analysis of pre-replicative complex genes in ALT development, Figure S2: representative images of PML and TERT immunostaining degrees of human brain tumor cases, Table S1: Differentially expressed genes revealed by transcriptome analysis, Table S2: Pathway analysis of RNA-Seq identifies a class of genes altered in zebrafish brain tumors with different TMMs, Table S3: Classification of DEG identified in brain tumor models based on TelNet specific genes functions, Table S4: List of genes in the “Activation of the pre-replicative complex” Reactome pathway, Table S5: list of oligo primers used for qPCR, Table S6: Intensity values of dot blot signals for c-circles in a panel of twenty human pediatric brain tumours.
