# Supplementary material for: Changes in the Expression of Pre-Replicative Complex Genes in hTERT and ALT Pediatric Brain Tumors
Source: Cancers (Basel). 2020 Apr 22;12(4):1028. doi: 10.3390/cancers12041028 (PMC7226177; doi:10.3390/cancers12041028)
Supplement: Supplementary file 1 [file cancers-12-01028-s001.zip › supplementary files/Supplementary Figures Cancers 260320.pdf]

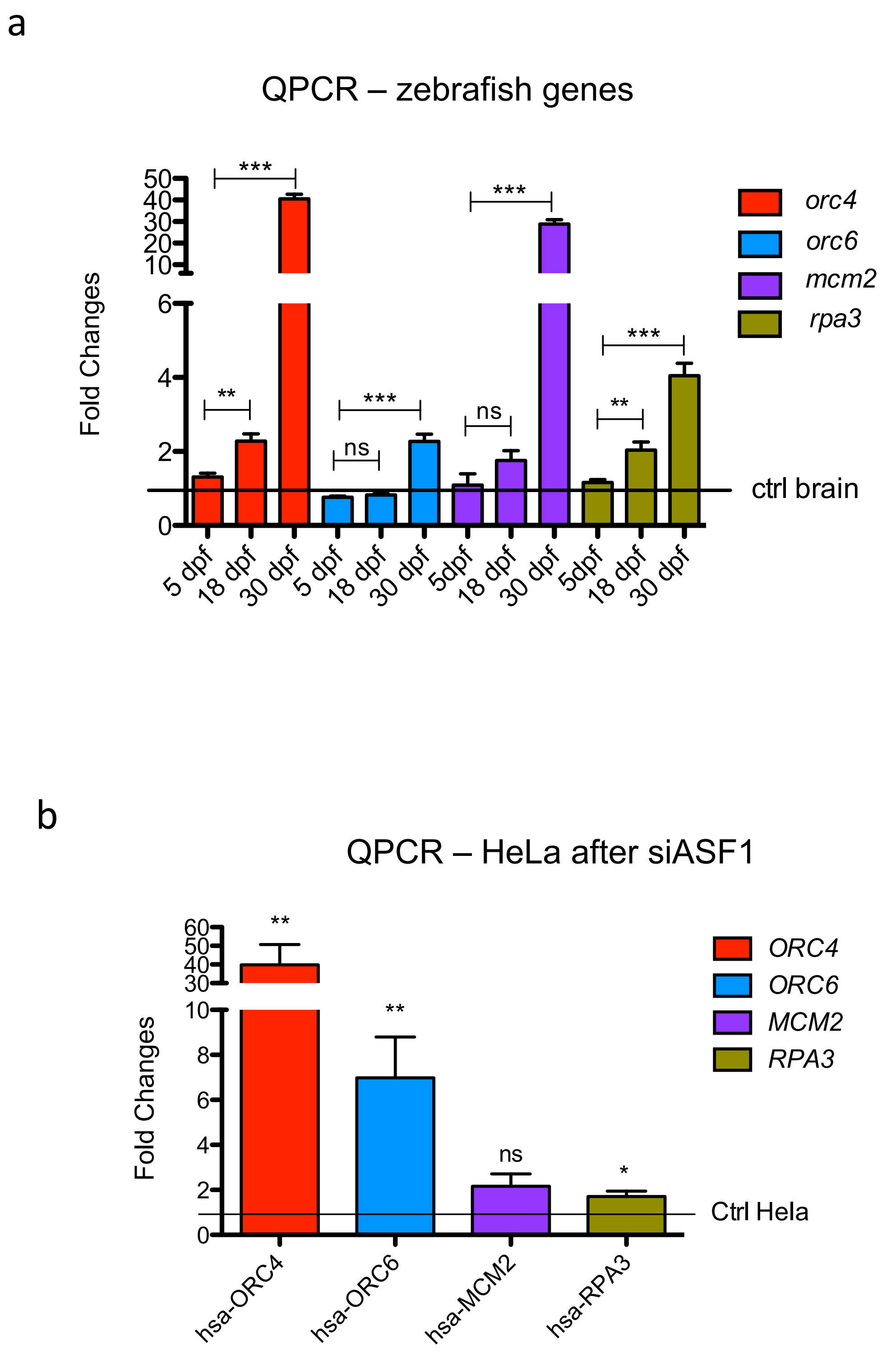

Figure S1: qPCR quantification of pre-replicative complex gene expression in zebrafish brain tumor development (**a**) and in HeLa cells treated with siASF1 (**b**). Gene expression is calculated relative to RSP11 (zf) or GAPDH (hsa) expression and to control brain (zf) or untreated HeLa cells (hsa). Error bars indicate means of at least 3 experiments ±SDs. dpf: day postfertilization. * p= 0.01 **p= 0.002 ***p<0.0001; ns= not significant

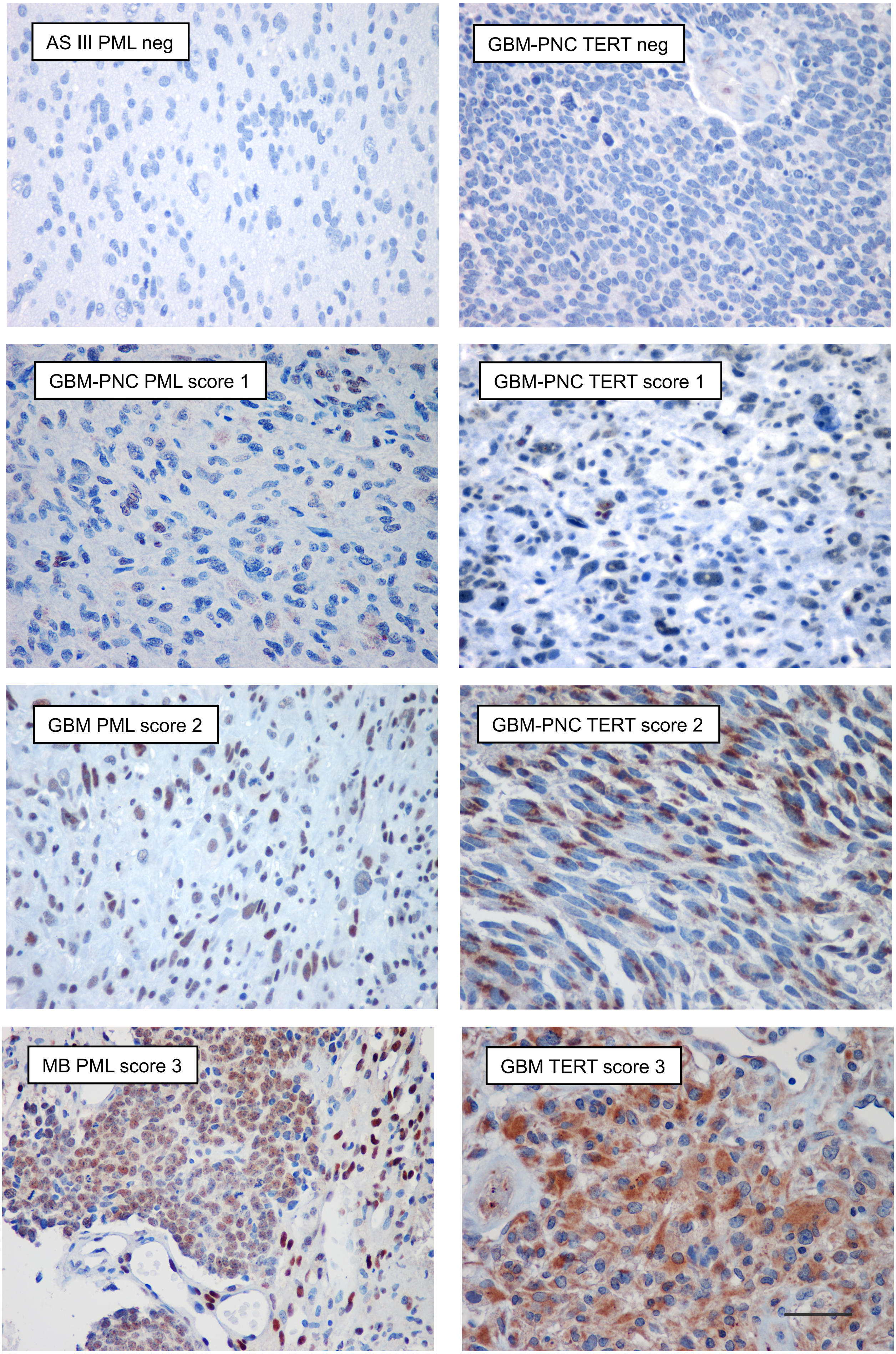

Figure S2: Representative images of PML and TERT immunostainings of human brain tumor cases (as indicated), showing examples used for the classification in table 1. Calibration bar: 50 μm
