# Supplementary material for: Changes in the Expression of Pre-Replicative Complex Genes in hTERT and ALT Pediatric Brain Tumors
Source: Cancers (Basel). 2020 Apr 22;12(4):1028. doi: 10.3390/cancers12041028 (PMC7226177; doi:10.3390/cancers12041028)
Supplement: Supplementary file 1 [file cancers-12-01028-s001.zip › supplementary files/Table S3.pdf]

Table S3

TelNet functions	DE genes	% TelNet DE genes per listed function
CHROMATIN ORGANIZATION	SGO1, HIST1H4A, EZH1, KMT2C, DPY30, C17orf49, HMGN2, HMGB2	36,36
DNA REPLICATION	CDC45, GAPDH, ORC4, RPA3, RECQL5, LIG1, MCM2, ORC6	36,36
TELOMERASE ACTIVITY	DHX36, TFAP2C, DCK	13,64
TERRA	ORC4, ORC6	9,09
DNA RECOMBINATION	RPA3, LIG1	9,09
PROTEIN SYNTHESIS	FKBP5, UCHL1	9,09
CHROMATIN STRUCTURE	HIST1H4A	4,55
CHROMATID COHESION	SGO1	4,55
ALT ASSOCIATED PML NUCLEAR BODIES	PIAS1	4,55

Table S3: Classification of DEG identified in brain tumour models based on TelNet specific genes functions.

The table reports the most representative telomere related-functions of differentially expressed genes revealed by transcriptome analysis between RAS-tert and RAS tumors, found in the TelNet database (http://www.cancertelsys.org/TelNet/). 51 genes were reported into the relative TelNet functions categories, The percentage identified the the most representative functions impaired.
