# Supplementary material for: Changes in the Expression of Pre-Replicative Complex Genes in hTERT and ALT Pediatric Brain Tumors
Source: Cancers (Basel). 2020 Apr 22;12(4):1028. doi: 10.3390/cancers12041028 (PMC7226177; doi:10.3390/cancers12041028)
Supplement: Supplementary file 1 [file cancers-12-01028-s001.zip › supplementary files/Table S4.pdf]

Table S4

Activation of the pre-replicative complex<br>Homo sapiens R-HSA-68962	
**UniProt**	**Genes**
Q9UBD	ORC3
O43913	ORC5
O43929	ORC4
Q13416	ORC2
Q9UJA3	MCM8
Q9Y5N6	ORC6
Q13415	ORC1
Q99741	CDC6
Q9H211	CDT1
P25205	MCM3
P33991	MCM4
P33992	MCM5
Q14566	MCM6
P33993	MCM7
P49736	MCM2
O75496	GMNN
P56282	POLE2
Q07864	POLE
Q9NRF9	POLE3
Q9NR33	POLE4
Q9UBU7	DBF4
O00311	CDC7
Q7L590	MCM10
P24941	CDK2
O75419	CDC45
Q13156	RPA4
P15927	RPA2
P35244	RPA3
P27694	RPA1
P49642	PRIM1
P09884	POLA1
Q14181	POLA2
P49643	PRIM2

Table S4: List of genes in the "Activation of the pre-replicative complex" Reactome pathway

List of the genes of the: Activation of the pre-replicative complex pathways - Homo sapiens, R-HSA-68962, identified by Reactome analysis using the 366 differentially expressed genes between RAS-Tert versus RAS tumours. This list of genes was used to analyse the activation of the pathway, in paediatric brain tumors data retrieved from pedCbioPortal (https://pedcbioportal.org/login.jsp). UniProt Genes code and gene names are reported.
