# Supplementary material for: Changes in the Expression of Pre-Replicative Complex Genes in hTERT and ALT Pediatric Brain Tumors
Source: Cancers (Basel). 2020 Apr 22;12(4):1028. doi: 10.3390/cancers12041028 (PMC7226177; doi:10.3390/cancers12041028)
Supplement: Supplementary file 1 [file cancers-12-01028-s001.zip › supplementary files/Table S5.pdf]

Table S5

Oligo primers used in qPCR	
**Name**	**Sequence**
ZF-orc4-Fw	AGC TCA GTT TGC CTC AGG AT
ZF-orc4-Rv	ACA CAC GAC TCA CAG CCA TA
ZF-orc6-Fw	CAT CTT TGA CCG GCT CTG TG
ZF-orc6-Rv	CTT GGG AGA CGT CAG AGG TT
ZF-mcm2-Fw	CTC TTT CAC GCG TCA AAC CA
ZF-mcm2-Rv	GCC TTG AAC AGA AGC TCG AG
ZF-rpa3-Fw	ACT GCT TGG CGT CTA GTC TT
ZF-rpa3-Rv	TTC GTC CAG GGG TTC ATT GA
ZF-rsp11-FW	ACAGAAATGCCCCTTCACTG
ZF-rsp11-Rv	GCCTCTTCTCAAAACGGTTG
HSA-ORC4-Fw	ACC TGC AGA GTT TCC AGA CA
HSA-ORC4-Rv	GCT ACA CAG TTG GCT TGC TT
HSA-ORC6-Fw	ACT TCT GCT GCA CTG CTT TC
HSA-ORC6-Rv	CTC CAT TTC CTT TGC TGG GG
HSA-MCM2-Fw	CTA CCT TTC ATT CCG GCG TG
HSA-MCM2-Rv	TGC AGA GAG GTT GTG GAT GT
HSA-RPA3-Fw	TCG TAG GGA GGC TGG AAA AG
HSA-RPA3-Rv	CCT TGG CGG TTA CTC TTC CA
HSA-ORC6-Fw	CTC CAT TTC CTT TGC TGG GG
HSA-GAPDH-Fw	ACA TCG CTC AGA CAC CAT
HSA-GAPDH-Rv	TGT AGT TGA GGT CAA TGA AGG G
