# Supplementary material for: Changes in the Expression of Pre-Replicative Complex Genes in hTERT and ALT Pediatric Brain Tumors
Source: Cancers (Basel). 2020 Apr 22;12(4):1028. doi: 10.3390/cancers12041028 (PMC7226177; doi:10.3390/cancers12041028)
Supplement: Supplementary file 1 [file cancers-12-01028-s001.zip › supplementary files/Table S6.pdf]

Table S6		
Sample #	(+ϕ29)-(-ϕ29) a.u.	CCircle dot blot + or -
1	1,384	-
2	3,034	+
3	1,755	-
4	1,88	-
5	5,393	+
6	9,988	+
7	5,431	+
8	0,196	-
9	0,647	-
10	4,671	+
11	0,486	-
12	3,119	+
13	4,072	+
14	0	-
15	10,28	+
16	7,754	+
17a	8,95	+
17b	0,599	-
18a	16,929	+
18b	1,786	-
19a	7,258	+
19b	1,65	-
20a	3,98	-
20b	2,23	-

Table S6: Intensity values of dot blot signals for c-circles in a panel of twenty human pediatric brain tumours (related to Table 1 and figure 4).

The table reports the intensity levels of c-circles of the 20 brain tumors (sample numbers in column 1 are the same as in table1) analysed by dot blot. Intensity values were calculated by Image Lab software, and expressed in arbitrary units (a.u.). The values were calculated after subtracting the background intensity and the intensity values of the same samples processed without phi29 (second column). Values above 2 are considered positive. The third column reports the final outcome (+ or -) reported in figure 4 and in table 1.
