# Supplementary material for: Changes in the Expression of Pre-Replicative Complex Genes in hTERT and ALT Pediatric Brain Tumors
Source: Cancers (Basel). 2020 Apr 22;12(4):1028. doi: 10.3390/cancers12041028 (PMC7226177; doi:10.3390/cancers12041028)
Supplement: Supplementary file 1 [file cancers-12-01028-s001.zip › supplementary files/Legend supl. table1 and 2.pdf]

Legends to Supplementary Table 1 and 2

Table S1: (Related to Fig.1: Differentially expressed genes revealed by transcriptome Analysis).

Analysis of RNA-Seq showed a class of genes altered in zebrafish brain tumors with different TMMs.) The table reports counts normalization to Trimmed Mean of M-values (TMM) revealed by transcriptome analysis in RAS-Tert versus RAS zebrafish brain tumors. 366 differentially expressed genes (DEG) were idendified using DESeq2, considering adjusted P- value <0.05 and a log2 fold change greater than 1 or smaller than -1. Ensemble ID, gene names and description are reported together with baseMean, log2FoldChange, log2FoldChange (lfcSE), pvalue and p adjusted value (padj). The human orthologous of 296 DE genes are also listed with their ensemble ID (Hs_ensembl_gene_) and human gene name.

Table S2: (Related to Fig.1: Reactome pathways analysis).

Analysis of RNA-Seq showed a class of genes altered in zebrafish brain tumors with different TMMs.) Pathway analysis performed with Reactome; enriched pathways are listed according to p-value and p adjusted value. The name of the pathways and the identification code are reported, together with the names and the number of DE genes identified by transcriptome analysis in RAS-Tert versus RAS zebrafish brain tumors.

Supplementary Tables

Table S3

**TelNet functions**	**DE genes**	**% TelNet DE genes per listed function**
CHROMATIN ORGANIZATION	SGO1, HIST1H4A, EZH1, KMT2C, DPY30, C17orf49, HMGN2, HMGB2	36,36
DNA REPLICATION	CDC45, GAPDH, ORC4, RPA3, RECQL5, LIG1, MCM2, ORC6	36,36
TELOMERASE ACTIVITY	DHX36, TFAP2C, DCK	13,64
TERRA	ORC4, ORC6	9,09
DNA RECOMBINATION	RPA3, LIG1	9,09
PROTEIN SYNTHESIS	FKBP5, UCHL1	9,09
CHROMATIN STRUCTURE	HIST1H4A	4,55
CHROMATID COHESION	SGO1	4,55
ALT ASSOCIATED PML NUCLEAR BODIES	PIAS1	4,55

Table S3: Classification of DEG identified in brain tumor models based on TelNet specific genes functions. (Related to Fig.1: Analysis of RNA-Seq showed a class of genes altered in zebrafish brain tumors with different TMMs.)

The table reports the most representative telomere related-functions of differentially expressed genes revealed by transcriptome analysis between RAS-Tert and RAS tumors, found in the TelNet database (http://www.cancertelsys.org/TelNet/). 51 genes were reported into the relative TelNet functions categories. The percentage identified the most representative functions impaired.

Table S4

Activation of the pre-replicative complex<br>Homo sapiens R-HSA-68962	
**UniProt**	**Genes**
Q9UBD	ORC3
O43913	ORC5
O43929	ORC4
Q13416	ORC2
Q9UJA3	MCM8
Q9Y5N6	ORC6
Q13415	ORC1
Q99741	CDC6
Q9H211	CDT1
P25205	MCM3
P33991	MCM4
P33992	MCM5
Q14566	MCM6
P33993	MCM7
P49736	MCM2
O75496	GMNN
P56282	POLE2
Q07864	POLE
Q9NRF9	POLE3
Q9NR33	POLE4
Q9UBU7	DBF4
O00311	CDC7
Q7L590	MCM10
P24941	CDK2
O75419	CDC45
Q13156	RPA4
P15927	RPA2
P35244	RPA3
P27694	RPA1
P49642	PRIM1
P09884	POLA1
Q14181	POLA2
P49643	PRIM2

Table S4: List of genes in the "Activation of the pre-replicative complex" Reactome pathway.

(Related to Fig.2: Analysis of RNA-Seq showed a class of genes altered in human brain tumors with different TMMs.)

List of the genes of the: Activation of the pre-replicative complex pathways - Homo sapiens, R-HSA-68962, identified by Reactome analysis using the 366 differentially expressed genes between RAS-Tert versus RAS

tumors. This list of genes was used to analyse the activation of the pathway, in paediatric brain tumors data retrieved from pedCbioPortal (https://pedcbioportal.org/login.jsp). UniProt Genes code and gene names are reported.
